# Psychometric analysis of the body responsiveness questionnaire in the Portuguese population

**DOI:** 10.1038/s41598-023-50530-5

**Published:** 2023-12-27

**Authors:** Sandra Torres, Ana Isabel Vieira, Filipa Mucha Vieira, Leonor Lencastre, Marina Prista Guerra, Kylee M. Miller, Maria Raquel Barbosa

**Affiliations:** 1 Faculty of Psychology and Education Sciences, University of Porto, Rua Alfredo Allen, 4200-135 Porto, Portugal; 2 Center for Psychology at the University of Porto, Rua Alfredo Allen, 4200-135 Porto, Portugal; 3 Institute on Development & Disability, Child Development and Rehabilitation Center (CDRC), Oregon Health and Science University, 901 E. 18th Ave, Eugene, OR 97403 USA

**Keywords:** Psychology, Health care

## Abstract

Body responsiveness refers to the tendency to be attuned to the body’s needs and use interoceptive information to guide behavior. Despite its potential beneficial effect on the development of positive body image, this construct is currently understudied. To boost research in this area, we examined the factor structure, gender invariance, and psychometric properties of a Portuguese translation of the Body Responsiveness Questionnaire (BRQ). A total of 650 men and women (aged 18–80 years) completed the Portuguese BRQ. To assess its convergent validity, participants also completed measures of body appreciation, emotion regulation, depression symptoms, and psychological quality of life. Exploratory factor analysis indicated a two-factor structure of the BRQ, which was upheld using confirmatory factor analysis: “Importance of Interoceptive Awareness” (ω = .85–.87) and “Perceived Connection” between body and mind (ω = .71–.74). BRQ scores had partial scalar invariance across gender, and no significant gender differences. Convergent and known-groups validity was supported. Participants with overweight/obesity (vs. normal weight) and middle-aged adults (vs. young adults) assigned higher importance to body signals to guide behavior. The Portuguese version of the BRQ is a psychometrically sound measure of body responsiveness and it may contribute to a comprehensive assessment of positive body image to guide intervention.

## Introduction

Body image, defined as the perceptions, thoughts, and feelings about one’s body^1^, has received research attention, especially regarding its negative aspects. Poor body image has been considered a risk factor for emotional health problems, such as depression^2^ and difficulties in emotion regulation^3^, and has been associated with low quality of life^4^.

Despite the primary focus on disturbance, contemporary research attention has turned toward positive body image (PBI). Considered a distinct and independent construct, PBI involves multiple facets, including body appreciation (gratitude for the function, health, and unique features of the body), body acceptance and love, adaptive investment in appearance and body care, broad conceptualization of beauty, inner positivity, and protective filtering against messages that threaten one’s body image^5^.

Research in this field has flourished over the last decade, fueled by the need for a more comprehensive view of body image, but also motivated by the encouraging findings that link PBI to several positive psychological indices, such as quality of life^6^ and emotional, psychological, and social well-being^7^. At the same time, some measures to assess PBI have been developed to facilitate this study, focusing on particular facets such as body appreciation (Body Appreciation Scale-2; BAS-2^8^), body functionality (Functionality Appreciation Scale; FAS^9^) and broad conceptualization of beauty (Broad Conceptualization of Beauty Scale; BCBS^10^). So far, body appreciation (using the BAS-2) is the most studied component of PBI. Although the body appreciation concept allows for a broader perspective of the experience of body image^8^, the investigation of other PBI dimensions is of major interest to achieve a holistic understanding of the construct.

Body responsiveness is one such understudied construct that is theoretically interwoven with PBI. Self-care behaviors that support PBI involve listening to the body’s needs and engaging in adaptive behaviors to attend to them^11^. Body responsiveness lies in the domain of body awareness but expands it into two aspects: the value placed in interoceptive information (i.e., internal bodily signals) to regulate behavior, and the connection between psychological and physical states. It is defined by the tendency to be attuned to the body’s needs and use embodied information to guide behavior^12^ and, as such, it is considered an essential aspect of embodiment^13^.

To assess this construct, Daubenmier^12^ developed the Body Responsiveness Questionnaire (BRQ). The original version of this 7-item questionnaire was first applied to diverse samples of American women (yoga, aerobic, nonyoga/nonaerobic practitioners, and undergraduate students) and revealed adequate internal consistency in all groups (Cronbach *α* = 0.70–0.83). In addition, body responsiveness was associated with lower levels of self-objectification, greater body satisfaction and body awareness, and less disordered eating attitudes. The factor structure of the original version was examined in a sample of participants who practice meditation^14^ and women with overweight/obesity^15^ and suggested the presence of two factors: “Importance of Interoceptive Awareness” (BRQ-Importance; composed by the four positively-keyed items), which assesses the importance of using interoceptive information to guide behavior (e.g., “I am confident that my body will let me know what is good for me”), and “Perceived Disconnection” (BRQ-Perceived Disconnection; composed by the three reversed items), which assesses the extent of disconnection between body and mind (e.g., “My mind and my body often want to do different things”).

To the best of our knowledge, only three studies have analyzed the factor structure and psychometric properties of the BRQ across other linguistic and national groups, in particular, in Hungary^13^, Germany^16^, and Colombia^17^. Using exploratory factor analyses (EFA), these studies confirmed the two-factor structure of the BRQ across distinct samples: community^13^, chronic pain patients^16^, and resident physicians^17^. Acceptable internal consistency was also reported, with Cronbach's *α* coefficients ranging between 0.75 and 0.83 for the BRQ-Importance factor and between 0.63 and 0.72 for the BRQ-Perceived Disconnection factor^13,16^. Additionally, evidence of convergent validity was provided by positive associations with body awareness, positive affect, spirituality, body-mind practice^13^, self-acceptance, and mindfulness^16^, as well as negative associations with negative affect, depression and stress symptoms^16^, and physical symptoms such as headache, stomachache, feeling tired and trouble sleeping^13^.

These results encourage cross-cultural research on the BRQ in order to extend the study of this construct within the scope of PBI. In Portugal, there are a lack of measures assessing the multiple aspects of body image and, to date, no instrument is available to assess body responsiveness. Therefore, the purpose of this study was to examine the factor structure, internal consistency, and validity (convergent, discriminant, and known-groups) of a Portuguese version of the BRQ in a community sample of Portuguese adults. In addition, we sought to investigate the measurement invariance of the BRQ across gender. Although research suggests higher levels of the BRQ-Perceived Connection factor in men compared to women^16^, this group comparison is only valid if the BRQ is invariant across gender. It is important to determine whether observed differences in scale scores across gender could be attributable to differential item functioning or are due to a real difference^18^.

Based on the literature reviewed above, we hypothesized that the BRQ would yield a two-factor structure and its items would be internally consistent^13,15–17^. We expected that the BRQ would be invariant across gender. Constructs used in the analysis of convergent validity were selected based on theoretical and empirical foundations of body responsiveness correlates. From an embodiment perspective (i.e., the experience of connection with the body), the accurate detection and interpretation of bodily cues confers benefits in terms of physical, mental, and social well-being^19,20^ and facilitates the access to important mechanisms of emotion regulation^21,22^. Thus, we predicted that BRQ scores would be negatively associated with depression symptoms^16^ and difficulties in emotion regulation. There is also some evidence that body awareness has been associated with health-related quality of life, in particular, mental health^20^. For this reason, we also hypothesized that the BRQ would be positively correlated with psychological quality of life. Lastly, we anticipated that BRQ scores would be positively associated with body appreciation^23^. Known-groups validity, that is, the instrument's ability to distinguish among distinct groups, was tested by comparing BRQ scores in weight categories subsamples (normal weight vs. overweight/obesity) and age groups (young adult group vs. middle-aged group vs. older group). These comparisons were informed by prior research indicating poorer overall interoception in individuals with overweight/obesity^24^, as well as highlighting the tendency for specific interoceptive sensibility (i.e., the tendency to attend or attempt to listen to specific internal body signals) to increase with advancing age^25^.

## Method

### Participants

The sample of this study consisted of 650 Portuguese adults (women *n* = 478, 73.5%, men *n* = 172, 26.5%). Participants ranged in age from 18 to 80 years (*M* = 31.91, *SD* = 13.79), comprising 415 young adults (18–30 years; 63.8%), 139 middle-aged adults (31–50 years; 21.4%) and 96 older adults (51–80 years; 14.8%). Participants ranged in BMI from 17.19 to 37.34 kg/m^2^ (*M* = 23.61, *SD* = 3.39). The prevalence of underweight was 2.5% (*n* = 16), normal weight 68.3% (*n* = 444), overweight 25.1% (*n* = 163), and obesity 4.2% (*n* = 27). The average years of schooling was 14.10 years (*SD* = 3.16, range 4–30 years).

### Measures

#### Demographics

Participants provided demographic information, including gender, age, years of schooling, as well as height and weight. The age variable was stratified into three groups: young adult group (18–30 years), middle-aged group (31–50 years), and older group (51–80 years). Self-reported height and weight were used to calculate the participant’s BMI (kg/m^2^). Weight status categories were created based on the World Health Organization (WHO) BMI classification^26^: underweight (BMI < 18.5), normal weight (BMI 18.5–24.9), overweight (BMI 25–29.9), and obesity (BMI ≥ 30).

#### Body responsiveness

Participants completed a Portuguese version of the BRQ^12^. The seven items were answered on a 7-point Likert-type scale, ranging from 1 (*not at all true about me*) to 7 (*very true about me*). The total score was computed as the mean of all items, with higher scores reflecting greater body responsiveness.

The BRQ’s translation process was based on the back-translation technique^27^. First, two psychology professors with expertise in body image independently translated the questionnaire into Portuguese. A consensus version of the two translations was obtained through a discussion with a research-team member with similar expertise. Second, the consensus version was backward translated into English by a bilingual psychologist (English mother tongue) that was not part of the research team. Third, the original BRQ and the back-translation versions were compared and minor differences between them were discussed and resolved by the two translators involved in the study. No major translational issues were identified.

#### Body appreciation

Body appreciation was measured by the Body Appreciation Scale-2 (BAS-2^8^; Portuguese version Lemoine et al.^28^; Meneses et al.^29^). The BAS-2 is composed of 10 items (e.g., “I respect my body”), which assess acceptance, respect, and care for one’s body and protection of one’s body from unrealistic beauty standards. All items were rated on a 5-point Likert-type scale, ranging from 1 (*never*) to 5 (*always*). The total score was computed as the mean of all items, with higher scores reflecting greater body appreciation. Psychometric studies with the Portuguese version of the BAS-2^28,29^ support the one-factor model, gender invariance, convergent validity, and adequate internal consistency (*α* between 0.91 and 0.94). In the present study, Cronbach’s *α* was 0.93.

#### Difficulties in emotion regulation

Difficulties in emotion regulation were assessed using the Difficulties in Emotion Regulation Scale (DERS^30^; Portuguese version Coutinho et al.^31^). This is a 36-item self-report measure rated on a 5-point Likert-type scale, ranging from 1 (*almost never*) to 5 (*almost always*) across six dimensions: (1) limited access to emotion regulation strategies, (2) non-acceptance of emotional responses, (3) lack of emotional awareness, (4) impulse control difficulties, (5) difficulties engaging in goal-directed behavior, and (6) lack of emotional clarity. It is possible to obtain a total score (sum of all items) and a score for each dimension; in this study we only used the total score. Higher total scores indicate greater difficulties in emotion regulation. This scale has shown high values for Cronbach's* α* in the original study, *α* = 0.93^30^, and for the Portuguese population, *α* = 0.93^31^. In this study, Cronbach's *α* was 0.95.

#### Depression symptoms

Depression symptomatology was assessed by the depression subscale of the Depression, Anxiety and Stress Scale (DASS^32^; Portuguese version Pais-Ribeiro et al.^33^). Participants were asked to refer to the previous week and provide their answers on a 4-point Likert-type scale, ranging from 0 (*did not apply to me at all*) to 3 (*applied to me very much, or most of the time*). Items were summed, with higher scores indicative of higher depressive symptomatology. The study of the Portuguese version confirmed the adequate psychometric properties of the DASS^33^. Here, the internal consistency coefficient for depression subscale scores on the DASS was good (*α* = 0.90).

#### Psychological quality of life

The WHO Quality of Life Scale Abbreviated Version (WHOQOL-Bref^34^; Portuguese version Vaz Serra et al.^35^) is a 26-item self-report questionnaire assessing four areas of quality of life: (1) physical, (2) psychological, (3) social, and (4) environment. Only items comprising the psychological domain were applied in the present study. Participants rated items on a 5-point Likert-type scale that measured intensity, frequency, evaluation, and capacity. In the study of the Portuguese version, Cronbach's *α* of the psychological domain was 0.84^35^. In the present study, the WHOQOL-Bref also demonstrated good internal consistency for this domain (*α* = 0.86).

### Procedure

Permission to use the BRQ in the present study was granted by the author of the original version (Jennifer J. Daubenmier, Ph.D.). Inclusion criteria to participate in the study were age ≥ 18 years (any gender) and Portuguese native speaker. Participants completed an online survey and were recruited via advertisements placed on social media sites, which was supplemented using a snowball sampling method. Upon entering the survey, participants read the information sheet and provided consent. Participation was voluntary and respondents did not receive monetary or in-kind rewards.

### Statistical analyses

Following the recommendations for test adaptation of body image measures^36^, we examined the factor structure of the Portuguese BRQ using a two-step analytic strategy consisting of EFA followed by CFA. Thus, the total sample was randomly split using the IBM SPSS Statistics version 28, resulting in a first split-half for EFA (women *n* = 236, men *n* = 92) and a second split-half for CFA (women *n* = 242, men *n* = 80). A priori sample size calculation^37^ revealed that for an effect size of 0.5 to be detected with 90% power at a 5% significance level, a minimum of 138 participants was required in the CFA. The total sample included no instances of missing data because completion of the online version of the instruments required a response to all items.

With data from the first split-half, we performed a principal-axis EFA in IBM SPSS Statistics version 28. The Kaiser–Meyer–Olkin test (KMO) and Bartlett's test of sphericity were conducted to determine the adequacy of the EFA. Adequate KMO values should be ≥ 0.60^38^, and Bartlett's test of sphericity should be significant. For the EFA, direct oblimin rotation was used. Factor extraction was based on the results of parallel analysis^39^, in which the factors in the current data were only retained if their eigenvalues were greater than the eigenvalues from the random data^39^. Item retention was based on Stevens' recommendations^40^, i.e., only items with factor loadings ≥ 0.40 were retained.

With data from the second split-half, we performed a CFA using the IBM SPSS AMOS version 28. Examination of data for normality indicated that the distributions were multivariate non-normal. Then, according to Kline^41^, maximum likelihood (ML) estimation with bootstrapping was performed to provide estimations of standard errors with accompanying confidence intervals (CI; bias-corrected at the 95% confidence level) and *p* values. Following the recommendations of Hu and Bentler^42^, we also used several fit indices, including the ratio of chi-square to degrees of freedom (χ^2^/df), the comparative fit index (CFI), the Tucker-Lewis index (TLI), the standardized root mean squared residual (SRMR), and the root mean square error of approximation (RMSEA) with a 90% CI. Good model fit was indicated by values ≤ 3.00 for χ^2^/df, values close to or above 0.95 for CFI and TLI, values close to 0.08 for SRMR, and values close to 0.06 for RMSEA^41,42^.

Using the second split-half, a multi-group CFA was also conducted to test whether BRQ scores were invariant across gender for configural invariance (i.e., the number of latent variables and the pattern of loadings of latent variables on indicators are similar across gender, that is, the unconstrained model should fit the data well in women and men), metric invariance (i.e., the magnitude of the loadings is similar across gender), and scalar invariance (i.e., item loadings and item intercepts are similar across gender^41,43^). Since full measurement invariance is often difficult to obtain, it has been recommended to consider partial measurement invariance, which involves the application of procedures to free constraints that are not equivalent between groups^36,44^. Because the χ^2^ differences (∆χ^2^) between nested models are sensitive to sample size, it has also been suggested that the results should be interpreted with other indicators of invariance, such as ∆CFI and ∆RMSEA or ∆SRMR^45^. A ∆CFI < 0.01 is indicative of metric invariance^45^, and scalar invariance is supported when ∆CFI < 0.01 and ∆RMSEA < 0.015 or ∆SRMR < 0.030^46^, although Cheung and Rensvold^45^ suggest that ∆CFI < 0.01 is sufficient. If scalar or partial scalar invariance was evidenced, independent-sample *t*-tests were planned to compare the BRQ scores across gender.

As for the internal consistency of BRQ scores in both subsamples, JASP (Version 0.16.4) was used to calculate the McDonald's Omega reliability coefficient (ω). A value ≥ 0.70 was considered adequate^47^. Finally, in the CFA portion of the dataset, the convergent and discriminant validity was assessed using the Fornell–Larcker criterion^48^. Convergent validity was assessed by calculating the average variance extracted (AVE); values ≥ 0.50 for the AVE were considered adequate^49,50^. Discriminant validity was assessed by using the squared correlation (*r*^2^) between factors and values of AVEs. When *r*^2^ is lower than the factor’s AVE, discriminant validity is established^49,50^. In the total sample, convergent validity was estimated by means of Pearson’s correlations (*r*) between BRQ scores and body appreciation, difficulties in emotion regulation, depression symptoms, and psychological quality of life. To assess known-groups validity, we performed independent-sample *t*-tests to compare the BRQ scores in two subsamples: participants with normal weight (18.5 < BMI < 24.9) vs. overweight/obesity (BMI > 25.0). As a measure of effect size, we used Cohen's^51^ benchmarks, where *d* of 0.20 is small, 0.50 is medium, and 0.80 is large. Additionally, a one-way analysis of variance (ANOVA) was performed, followed by Gabriel post hoc tests, to discern variations in BRQ scores among the age groups (young adult group vs. middle-aged group vs. older group). Eta-squared (η^2^) was interpreted according to Cohen’s^51^ guidelines: 0.01 = small effect, 0.06 = medium effect, and 0.14 = large effect.

Data from the current study are available from the corresponding author (S.T.) upon reasonable request.

### Ethics approval

This study was performed in line with the principles of the Declaration of Helsinki. Approval was granted by the Ethics Committee of the Faculty of Psychology and Education Sciences, University of Porto (Reference 2018/12-6) and the Data Protection Unit of the University of Porto (Ref. 4/2020).

### Consent to participate

Informed consent was obtained from all individual participants included in the study.

## Results

### Exploratory factor analysis

In the subsample from the first split-half (*n* = 328), the KMO = 0.74 supported the sampling adequacy for the analysis. Bartlett's test of sphericity χ^2^ (21) = 828.13, *p* < 0.001, indicated that correlations between items were sufficiently large for EFA. Two factors had eigenvalues over Kaiser’s criterion of 1, and parallel analysis revealed that a two-factor structure should be extracted. We retained two factors, which explained 55.0% of the variance. Table 1 shows the factor loadings after rotation, which ranged from 0.55 (Item 4) to 0.89 (Item 6).

**Table 1:** Body responsiveness questionnaire items in English and Portuguese (in italics) and factor loadings derived from the exploratory factor analyses with the first split-half subsample (n = 328).

BRQ items/*Portuguese translation*	Rotated factor loadings	
	Factor 1	Factor 2
1. I am confident that my body will let me know what is good for me/*Estou confiante de que o meu corpo me dirá o que é bom para mim*	.59	
2. My bodily desires lead me to do things that I end up regretting (R)/*Os meus desejos corporais levam-me a fazer coisas das quais acabo por me arrepender*		.71
3. My mind and body often want to do two different things (R)/*A minha mente e o meu corpo querem, frequentemente, fazer coisas diferentes*		.80
4. I suppress my bodily feelings and sensations (R)/*Eu suprimo os meus sentimentos e sensações corporais*		.55
5. I “listen” to my body to advise me about what to do/*Eu “oiço” o meu corpo para que ele me aconselhe sobre o que fazer*	.72	
6. It is important for me to know how my body is feeling throughout the day/*É importante para mim saber como é que o meu corpo se está a sentir ao longo do dia*	.89	
7. I enjoy becoming aware of how my body feels/*Eu gosto de ir tomando consciência de como o meu corpo se sente*	.86	

*R* reversed items. Only factor loadings > .40 are presented.

In this subsample, internal consistency (McDonald’s ω) for factor 1 was 0.85, 95% CI [0.82, 0.88], and for factor 2 was 0.74, 95% CI [0.69, 0.78].

### Confirmatory factor analysis

**Figure 1:**
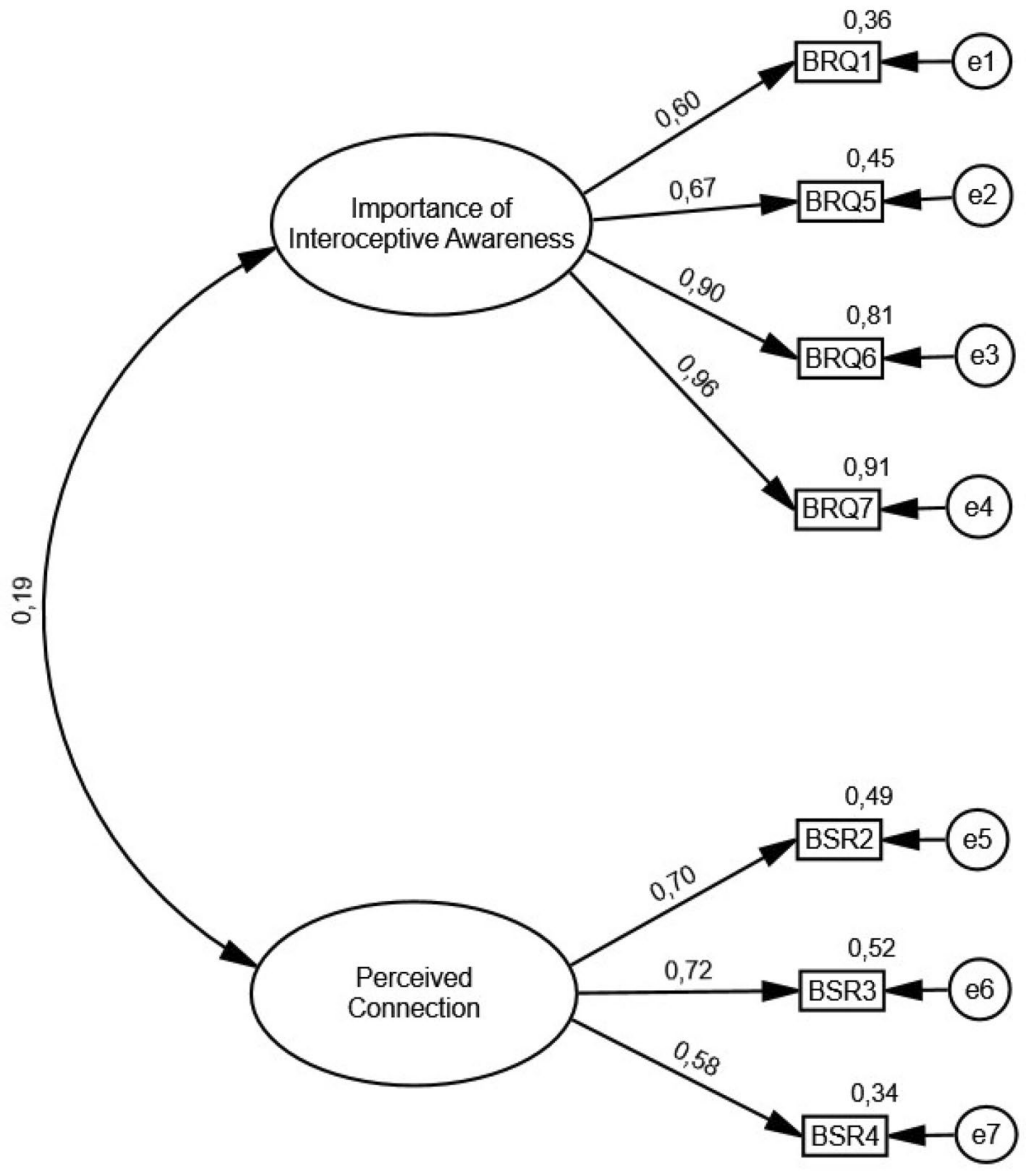
Path diagram and estimates for the two-factor model of body responsiveness questionnaire scores in the second split-half subsample (n = 322). *Note.* The path factor loadings are standardized. The ovals are the latent constructs, the rectangles are the measured variables, and the small circles are the residual variables (variances).

In the subsample from the second split-half (*n* = 322), CFA indicated that the two-factor model of BRQ scores had a good fit across all indices: χ^2^/df = 1.666, CFI = 0.991, TLI = 0.985, SRMR = 0.030, and RMSEA = 0.046 (90% CI 0.000, 0.078). The standardized estimates of factor loadings were all adequate (see Fig. 1 and Table 2). The items that cluster on the same factors suggest that factor 1 represents the “Importance of Interoceptive Awareness” (BRQ-Importance) and factor 2 represents the “Perceived Connection” (BRQ-Perceived Connection). Following the same procedure as Cramer et al.^16^, the factor 2 was slightly renamed from the English original in order to reflect that higher reverse-scored values represent higher body responsiveness.

**Table 2:** Standardized item-factor loadings for the body responsiveness questionnaire derived from the confirmatory factor analyses with the second split-half subsample (n = 322).

Items	Standardized 95% CI			
	Estimate	Lower	Upper	*p*
BRQ-importance^a^				
BRQ1	0.60	.48	.70	.005
BRQ5	0.67	.55	.75	.010
BRQ6	0.90	.84	.94	.005
BRQ7	0.96	.93	.98	.004
BRQ-perceived connection^a^				
BRQ2	0.70	.59	.84	.004
BRQ3	0.72	.59	.83	.005
BRQ4	0.58	.47	.72	.003

^a^Correlation between BRQ-importance and BRQ-perceived connection, *r* = .19 (95% CI = [.32, .01]), *p* = .04.

The convergent validity was acceptable for the BRQ-Importance factor, as AVE was greater than 0.50 (AVE = 0.63) and close to acceptable for the BRQ-Perceived Connection factor (AVE = 0.45). The discriminant validity was also supported considering that the square correlation between the two factors (*r*^2^ = 0.17) was lower than each factor's AVE (0.63 and 0.45). The low correlation between the two BRQ factors indicates that a score for each factor should be calculated individually, rather than an overall score.

In this subsample, internal consistency (McDonald’s ω) for the BRQ-Importance factor was 0.87, 95% CI [0.85, 0.89], and for the BRQ-Perceived Connection factor was 0.71, 95% CI [0.66, 0.77].

### Measurement invariance across gender

As reported in Table 3, the results of the multi-group CFA conducted in the subsample from the second split-half showed that the unconstrained latent model had adequate fit in women (*n* = 242) and men (*n* = 80) groups, suggesting configural invariance. BRQ is also metric invariant across women and men (metric ΔCFI = 0.008). However, the value for ΔCFI was above acceptable levels for scalar invariance (scalar ΔCFI = 0.011, scalar ΔRMSEA = 0.003, scalar ΔSRMR = 0.001). Therefore, possible sources of non-invariant loadings were explored. Item 4 was found to have the greatest difference between women and men. As such, we relaxed the equality constraint for its intercept, obtaining an adequate partial scalar invariance (see Table 3). Independent-sample *t*-tests showed that there were no gender differences in the BRQ-Importance factor (women *M* = 5.10, *SD* = 1.42; men *M* = 5.25, *SD* = 1.31), *t* (320) = − 0.86, *p* = 0.389, *d* = − 0.11, nor in the BRQ-Perceived Connection factor (women *M* = 4.50, *SD* = 1.67; men *M* = 4.17, *SD* = 1.57), *t* (320) = 1.57, *p* = 0.119, *d* = 0.20.

**Table 3:** Measurement invariance for the two-factor body responsiveness questionnaire model across gender in the second split-half subsample (n = 322).

Model	χ^2^	*df*	CFI	RMSEA	SRMR	Model comparison	∆χ^2^	∆CFI	∆RMSEA	∆SRMR	∆*df*	*p*
Configural	49.504	26	.976	.053	.090							
Metric	62.009	31	.968	.056	.096	Configural vs. metric	12.504	.008	.003	.006	25	.028
Scalar	80.204	38	.957	.059	.097	Metric vs. scalar	18.196	.011	.003	.001	7	.011
Partial scalar	70.333	37	.966	.053	.097	Metric vs. partial scalar	8.325	.002	.003	.001	6	.215

*CFI* comparative fit index, *RMSEA* root mean square error of approximation, *SRMR* standardized root mean square residual.

### Convergent and known-groups validity

The correlation coefficients between the BRQ factors and other study variables were all significant (*p* < 0.001) and are summarized in Table 4. The association with body appreciation was higher in the BRQ-Importance factor (*r* = 0.54) than in the BRQ-Perceived Connection factor (*r* = 0.31). Similar results were found regarding the psychological quality of life (BRQ-Importance factor: *r* = 0.49; BRQ-Perceived Connection factor: *r* = 0.27). Both BRQ factors correlated negatively with difficulties in emotion regulation (*r* = − 0.35 and − 0.40) and depression symptomology (*r* = − 0.29 and − 0.30), although the strength of the association with depression is somewhat smaller.

**Table 4:** Bivariate correlations between body responsiveness questionnaire factors and additional variables included in the study for the total sample (N = 650).

	1	2	3	4	5	6
1. BRQ-Importance	–	.11**	.54***	− .35***	− .29***	.49***
2. BRQ-Perceived Connection		–	.31***	− .40***	− .30***	.27***
3. Body Appreciation			–	.52***	− .48***	.67***
4. Difficulties in emotion regulation				–	.71***	− .59***
5. Symptoms of depression					–	− .60***
6. Psychological quality of life						–

*p < .05; **p < .01; ***p < .001.

Using the total sample, independent-sample *t*-tests also showed that participants with normal weight (*M* = 5.21, *SD* = 1.37) had higher scores on the BRQ-Importance factor than those with overweight/obesity (*M* = 4.86, *SD* = 1.33), *t* (632) = 2.99, *p* < 0.001, *d* = 0.26. There were no significant differences between the normal weight (*M* = 4.45, *SD* = 1.68) and overweight/obesity (*M* = 4.24, *SD* = 1.59) groups in the BRQ-Perceived Connection factor, *t* (632) = 1.46, *p* = 0.07, *d* = 0.13. ANOVA revealed significant differences among the age groups in the BRQ-Importance factor, *F* (2,647) = 6.02, *p* = 0.003, η^2^ = 0.02. Gabriel's post-hoc test showed that the middle-aged group (*M* = 5.47, *SD* = 1.37) had higher scores on the BRQ-Importance factor than the young adult group (*M* = 5.01, *SD* = 1.36). No differences were found between the young adult and older adult (*M* = 5.09, *SD* = 1.29) groups, nor between the middle-aged and older adult groups on the BRQ-Importance factor. ANOVA also revealed significant differences among the age groups in the BRQ-Perceived Connection factor, *F* (2,647) = 5.01, *p* = 0.007, η^2^ = 0.02. Gabriel's post-hoc test indicated that the older adult group (*M* = 4.85, *SD* = 1.60) had higher scores on the BRQ-Perceived Connection factor than the young adult group (*M* = 4.27, *SD* = 1.65). No differences were found between the young adult and middle-aged (*M* = 4.47, *SD* = 1.70) groups, nor between the middle-aged and older adult groups on the BRQ-Perceived Connection factor.

## Discussion

This study aimed to examine the factor structure and psychometric properties of the BRQ in Portuguese-speaking adults. In terms of factorial validity, using both the EFA and CFA, we found that scores on the Portuguese BRQ had a two-dimensional structure, which measures the importance of attending to bodily signals to guide behavior (BRQ-Importance) and the extent of the perceived connection between psychological and bodily states (BRQ-Perceived Connection). These results converge with both the parent study conducted with American adults^14,15^ as well as samples from other linguistic and cultural backgrounds^13,16,17^ and reinforce the use of the BRQ as a two-subscale measure of body responsiveness.

Across both split-half subsamples, BRQ factors yielded evidence of internal consistency (ω between 0.71 and 0.87). As observed in previous studies^13,16^, the BRQ-Perceived Connection factor had lower Omega coefficients probably due to a small number of items.

We examined the measurement invariance of the BRQ factors across gender. To our knowledge, this is the first study to provide evidence of gender invariance of the BRQ. It was found that scores on the Portuguese BRQ achieved configural and metric invariance but only partial scalar invariance. Our analysis of gender comparison did not find significant differences. This finding contrasts with the study by Cramer et al.^16^ carried out with the German version of the BRQ, in which the BRQ-Perceived Connection factor presented higher scores in men compared to women. However, it should be noted that the German BRQ study used a clinical sample of participants with chronic pain and did not test for invariance across gender.

Further, we were able to support the convergent and discriminant validity of the Portuguese BRQ. The BRQ-Importance factor showed an adequate AVE value and, despite this index being lower in the BRQ-Perceived Connection factor, it was close to the acceptable level. Discriminant validity was also suggested by AVE’s metrics, indicating that BRQ factors are distinct and uncorrelated.

Evidence of convergent validity was also supported by significant associations of the BRQ factors with indices of PBI and interoceptive awareness. The strong link between body responsiveness and body appreciation is in line with Oswald et al.’s study^23^ and lends credence to the view that being attentive to the body’s needs is an important boost to PBI. By valuing the body’s feedback, individuals are more prone to engage in mindful self-care behaviors and thereby reduce the focus on physical appearance. Self-care practices encourage the maintenance of physical and mental health and are also a way of loving and respecting the body^52^.

A robust body of research has suggested that interoceptive processes may play an important role in shaping health^53^. One of the explanatory mechanisms relates to emotion regulation. As bodily cues and sensations are a key source for emotional experience, when interoceptive awareness is poor, there is a reduced ability to interpret and regulate emotions and, therefore, an increased risk of developing emotional disorders, such as depression^54^. Based on these considerations, the negative association observed between the BRQ factors and difficulties in emotion regulation and depressive symptoms, as well as the positive association with psychological quality of life, support the BRQ’s convergent validity. In a similar vein, Tihanyi et al.^13^ and Cramer et al.^16^ have found a negative correlation between BRQ factors and negative affect and depression symptoms, respectively. It is noteworthy that psychological quality of life and the importance placed on bodily sensations for guidance are tightly interwoven, calling attention to the relevance of body responsiveness in the context of mental health.

Based on evidence that deficits in interoception were associated with higher BMI^24^, we compared participants with normal weight vs. overweight/obesity on BRQ scores. As hypothesized, participants with overweight/obesity attached less importance to body’s feedback, which provides preliminary evidence for the BRQ’s known-groups validity. Theoretically, low sensitivity to bodily cues can limit satiety from being strongly weighted into eating and food-related decision-making. On the other hand, as impaired interoception may interfere with emotion regulation, this difficulty may indirectly increase susceptibility to emotional overeating and, in turn, weight gain^55^. Conversely, being attentive to body signals can lead to an adaptive style of eating, such as intuitive eating, in which physiological hunger and satiety cues are used to make food choices^56^. Indeed, correlates of intuitive eating have included interoceptive accuracy^57^, interoceptive awareness, and responsiveness to bodily states^58^. It should be noted, however, that participants with overweight/obesity did not show lower body-mind connectedness relative to the normal-weight group. This was an unexpected finding that needs to be further explored. It is possible that body-mind disconnection is limited to interoception related to appetite/satiety, which is not specifically addressed in this BRQ factor.

Known-groups validity was also assessed by comparing BRQ scores across the three different age groups. Young adults assigned less importance to bodily sensations for guidance (relative to middle-aged adults) and perceived a lower connection between psychological and physical states (relative to older adults). These findings support the known-groups validity of the Portuguese BRQ and converge with the previous study by Tihanyi et al.^13^, which found a positive correlation between age and the BRQ factors among university students. With ongoing age, adults commonly redirect their former focus toward physical appearance, to placing increasing value on the health and functionality of their bodies^59^. The perception of greater vulnerability for health-related problems that typically arises with age can enhance individuals' attentiveness and familiarity with physiological signals^25^, thereby fostering a stronger mind–body connection.

Some strengths of the present study include the use of a two-step analytic strategy to determine the BRQ factor structure and the measurement of invariance across gender. However, our study is not without limitations. The first concerns the use of an online recruitment strategy, which prevents the results from being treated as representative of the Portuguese adult population. As most participants were from the North of Portugal, future works should include more participants from other regions. Second, we administered the measures in a fixed order and, as such, potential order effects were not controlled. Third, the design of our study did not allow for a second administration point and, therefore, the examination of test–retest reliability of BRQ scores. Further research will profit from investigating temporal stability and the inclusion of a broader range of variables to establish additional indices of construct validity, such as other PBI dimensions and reactions to body experiences (e.g., intuitive eating, sexual assertiveness). Finally, the small subsample of older adults prevented us from testing measurement invariance across age. This is an important topic for future research considering the putative age effect on self-perceived sensitivity to internal bodily sensations^25,60^.

Despite the limitations, the current study suggests that the Portuguese BRQ is a psychometrically valid tool for assessing responsiveness to bodily sensations. This measure can contribute to a more comprehensive assessment of body image by addressing an important aspect of embodiment that has been ignored by most of the body-related questionnaires. In addition, the link between the BRQ and emotion regulation and psychological quality of life observed in this study suggests that this measure can be a good indicator of health-promoting attitudes toward one's body-mind.

## Data Availability

The datasets analyzed during the current study are available from the corresponding author upon reasonable request.
